# Air Pollution, Neonatal Immune Responses, and Potential Joint Effects of Maternal Depression

**DOI:** 10.3390/ijerph18105062

**Published:** 2021-05-11

**Authors:** Jill Hahn, Diane R. Gold, Brent A. Coull, Marie C. McCormick, Patricia W. Finn, David L. Perkins, Sheryl L. Rifas Shiman, Emily Oken, Laura D. Kubzansky

**Affiliations:** 1 Department of Social and Behavioral Sciences, The Harvard T.H. Chan School of Public Health, Boston, MA 02115, USA; mmccormi@hsph.harvard.edu (M.C.M.); lkubzans@hsph.harvard.edu (L.D.K.); 2 Channing Division of Network Medicine, Brigham and Women’s Hospital, Harvard Medical School, Boston, MA 02115, USA; diane.gold@channing.harvard.edu; 3 Department of Environmental Health, Harvard T.H. Chan School of Public Health, Boston, MA 02115, USA; bcoull@hsph.harvard.edu; 4 Department of Biostatistics, Harvard T.H. Chan School of Public Health, Boston, MA 02115, USA; 5 Division of Pulmonary, Critical Care, Sleep, and Allergy, Department of Medicine, University of Illinois at Chicago, Chicago, IL 60612, USA; pwfinn@uic.edu; 6 Department of Microbiology and Immunology, University of Illinois at Chicago, Chicago, IL 60612, USA; 7 Division of Nephrology, Department of Medicine, University of Illinois at Chicago, Chicago, IL 60612, USA; perkinsd@uic.edu; 8 Department of Surgery, University of Illinois at Chicago, Chicago, IL 60612, USA; 9 Department of Bioengineering, University of Illinois at Chicago, Chicago, IL 60612, USA; 10 Division of Chronic Disease Research Across the Lifecourse, Department of Population Medicine, Harvard Medical School and Harvard Pilgrim Health Care Institute, Boston, MA 02215, USA; sheryl_rifas@hphc.org (S.L.R.S.); emily_oken@harvardpilgrim.org (E.O.)

**Keywords:** social determinants of health, chemical stressors, non-chemical stressors, air pollution, maternal prenatal depression, immune system, cord blood mononuclear cells, cytokines, intergenerational effects

## Abstract

Prenatal maternal exposure to air pollution may cause adverse health effects in offspring, potentially through altered immune responses. Maternal psychosocial distress can also alter immune function and may increase gestational vulnerability to air pollution exposure. We investigated whether prenatal exposure to air pollution is associated with altered immune responses in cord blood mononuclear cells (CBMCs) and potential modification by maternal depression in 463 women recruited in early pregnancy (1999–2001) into the Project Viva longitudinal cohort. We estimated black carbon (BC), fine particulate matter (PM_2.5_), residential proximity to major roadways, and near-residence traffic density, averaged over pregnancy. Women reported depressive symptoms in mid-pregnancy (Edinburgh Postnatal Depression Scale) and depression history by questionnaire. Immune responses were assayed by concentrations of three cytokines (IL-6, IL-10, and TNF-α), in unstimulated or stimulated (phytohemagglutinin (PHA), cockroach extract (Bla g 2), house dust mite extract (Der f 1)) CBMCs. Using multivariable linear or Tobit regression analyses, we found that CBMCs production of IL-6, TNF-a, and IL-10 were all lower in mothers exposed to higher levels of PM_2.5_ during pregnancy. A suggestive but not statistically significant pattern of lower cord blood cytokine concentrations from ever (versus never) depressed women exposed to PM_2.5_, BC, or traffic was also observed and warrants further study.

## 1. Introduction

A large body of research has demonstrated that exposure to air pollution can contribute to illness and death [1,2] and that these effects may be mediated by inflammatory mechanisms [3,4]. Inhalation of particulate air pollution causes a systemic inflammatory reaction; this has been identified as the underlying mechanism linking air pollution exposure to a higher risk of cardiovascular and other immune-mediated diseases [5]. Adult human and toxicologic studies have demonstrated that air pollution exposure may be associated with increased levels of circulating pro-inflammatory cytokines, including tumor necrosis factor-alpha (TNF-a) and interleukin-6 (IL-6) as well as a range of other cytokines [5].

Data are more limited on whether gestational pollution exposures may affect perinatal or infant immune function. Rodent models have demonstrated both increases [6] and decreases [7] in inflammatory responses in infant offspring of mothers exposed to fine (PM_2.5_, particles < 2.5 µm in diameter, composed of a complex mix including carbon, sulfates, and nitrates) and ultrafine (the fraction of PM_2.5_ < 0.1 µm diameter) particulate matter during pregnancy. A small number of human studies to date have observed alterations in distributions of neonatal cord blood immune cells with exposure to higher concentrations of traffic-related air pollutants [8], including decreases in the total T-cell fraction (CD3+) and in CD4+ T-helper cell and T-regulatory cell (CD4+/CD25+) fractions associated with increases in gestational ambient particle exposures [9,10]; however, the implications for later inflammatory responses are not known.

Environmental toxicants and adverse psychosocial exposures share common physiological pathways [11], and there is some evidence from animal [12] and human studies [13,14,15] that psychosocial stress may increase susceptibility to environmental exposures such as air pollution (termed “double jeopardy,” [16]). For example, a prospective study of Mexican women recruited in pregnancy (*n* = 552) found that higher exposure to particulate matter 2.5 microns or less (PM_2.5_) during the first trimester was associated with an increased risk of wheeze at age 4 only among children whose mothers reported high prenatal stress (defined by scoring above the median on an index of negative life events) [17]. In Project Viva, a cohort of pregnant women and their offspring recruited in eastern MA in 1999–2002, we previously showed a direct association between maternal depression and decreased concentrations of IL-10 secreted from cord blood lymphocytes [18]. Since depression is a recurring disease [19,20], and its health effects are considered a result of cumulative burden rather than of acute exposure [21], we defined women who reported a history of depression or who reported depression symptoms during pregnancy as having depression. Upon stimulation with cockroach allergen, dust mite allergen or PHA, levels of IL-10 were lower in cord blood from ever versus never depressed women (percentage difference: cockroach extract = −41.4, *p* = 0.027; house dust mite extract = −36.0, *p* = 0.071; PHA = −24.2, *p* = 0.333).

In the current study, conducted in the Project Viva cohort, we first sought to evaluate whether maternal exposure to air pollution during pregnancy was associated with alterations in offspring immune responses evident at birth. Following prior studies, we hypothesized that higher exposure to air pollution would be associated with higher levels of pro-inflammatory (IL-6 and TNF-α) and lower levels of anti-inflammatory (IL-10) cytokines. Increased cytokine production in response to immune stimulant exposure has been interpreted as a measure of the immune system’s competence; we, therefore, assessed CBMC cytokine production after stimulation with three immune stimulants [21]. We then tested the hypothesis that maternal prenatal depression would exacerbate the effects of air pollution exposure either additively or synergistically. Following prior work, we considered a range of factors that might be associated with the risk of exposure to air pollution and might also influence immune responses, including mother’s demographics and cigarette use.

## 2. Materials and Methods

### 2.1. Study Sample

Project Viva is a longitudinal pregnancy cohort initiated to examine how events during early development are associated with offspring health outcomes. The study has been described in detail elsewhere [22]. Briefly, from 1999–2002, pregnant mothers were enrolled at their first prenatal obstetric visit (median 9.9 weeks of gestation) at eight locations of the Atrius Harvard Vanguard Medical Association across urban (including Boston) and suburban Eastern Massachusetts. Women were eligible if they could answer questions in English, were less than 22 weeks pregnant at the time of enrollment, had a singleton pregnancy, and planned to remain in the study area through delivery. Mothers gave written informed consent upon enrollment, including cord blood collection at delivery and access to medical (and pharmacy) records. All participants delivered at one of two area hospitals, but only one hospital was willing to facilitate cord blood collection. Delivering clinicians collected venous umbilical cord blood from non-emergent deliveries at the participating hospital (*n* = 1029 of the 2128 live singleton births), among which we measured cytokines for 463 (See Figure S1 for sample flowchart). Study staff met with participants after routine appointments during the first and second trimesters and 1–3 days after delivery on the post-partum maternity floor. At each visit, study staff conducted a range of health and developmental assessments. Project Viva was approved by the Institutional Review Board of Harvard Pilgrim Health Care [22].

For each air pollution analysis, we excluded mother/infant pairs if they were missing data on that measure of air pollution. We additionally excluded the 4% of women who had their category of distance to a major road or near-residence traffic density changed during pregnancy (Table S1 compares those excluded from and included in the study).

### 2.2. Measures

#### 2.2.1. Air Pollution

We considered four measures of air pollution exposure during pregnancy: distance to major road, neighborhood traffic density, exposure to black carbon (BC), and exposure to PM_2.5_. These four air pollution measures are correlated [23] but also capture different types of traffic-related pollution. Distance to a major road and neighborhood traffic density are considered proxies for long-term traffic-related ambient air pollution exposure and encompass components of pollution that have not been measured individually. BC and PM_2.5_ exposure were calculated in this sample using validated, spatiotemporal LUR models [23,24] incorporating data collected from ambient monitors.

Distance to a major road: Mothers provided their residential address at each study visit. ArcGIS 10.1 and Street MapTM North America (both are products of ESRI, Redlands, CA, USA) were used to calculate the shortest distance in meters between the geocoded location of the mother’s reported residential address and a primary major roadway (US Census feature class A1 or A2). We used roadway proximity estimates for addresses reported at birth to capture exposure to traffic-related air pollution throughout the pregnancy. Because addresses reported at enrollment were available, it was possible to determine whether an individual moved during pregnancy, reducing concerns about misclassification of residential air pollution exposure. Although 11% of participants moved at some point during pregnancy, 96% did not change their category of roadway proximity from enrollment through the child’s birth. Because concentrations of traffic-related air pollution components decay exponentially with increasing distance from the source [25,26], following previous work in this cohort, we modeled the measure either as the natural log (ln) (proximity to a roadway) or categorized as: <100 m, 100–<200 m, 200–<400 m, and ≥400 m.

Near-residence traffic density: was defined as the length of all roads (km) within 100 m of the residence, multiplied by those roads’ traffic counts (vehicles/day). Data from the Massachusetts Department of Transportation was used to provide traffic count estimates for enrollment and birth addresses (Mass DOT 2002). Following previous studies with traffic density assessments [27,28], we used ln–transformed traffic density modeled as a continuous exposure, with density levels at birth used to capture exposure throughout pregnancy.

BC: We used a spatially and temporally resolved model based on daily BC measurements from 148 temporary and permanent monitors that operated in the region starting in January 1999 [29]. Other inputs included area land use, traffic density (average daily traffic (vehicles/day) × length of road (km) within 100 m), meteorological data, seasonal and long-term trend terms, and interactions between these variables [29,30]. Exposure was assigned only if participants resided in Massachusetts, where model predictions were valid, for at least 90% of the days in an exposure period (8 individuals were excluded for the BC analyses). BC exposure during pregnancy was calculated by averaging daily BC concentrations for each individual over the prenatal period, calculated as the date of last menstrual period through the day before birth [31], and was modeled as a continuous variable.

PM_2.5_: The PM_2.5_ model used mixed models with random slopes for day to calibrate aerosol optical depth (AOD) data from the Moderate Resolution Imaging Spectroradiometer aboard the Earth Observing System satellites with daily measured concentrations (2000–2008) of PM_2.5_ from the United States Environmental Protection Agency air quality monitoring networks across New England, at a 10 × 10 km spatial grid resolution [29,30]. A generalized additive mixed model with spatial smoothing was then used to estimate PM_2.5_ in location-day pairs with missing AOD, using regional measured PM_2.5_, AOD values in neighboring cells, and land use. “Out-of-sample” ten-fold cross-validation was used to quantify the accuracy of the model’s predictions. Models performed well both for days with (mean “out-of-sample” R^2^ = 0.87) and without (mean “out-of-sample” R^2^ = 0.85) available AOD data [23]. To estimate the PM_2.5_ exposure during pregnancy, the mother’s residence at delivery was linked to a 10 × 10 km grid point. Exposure was only assigned if a participant resided in New England, where model predictions were valid for at least 90% of the days in an exposure period (4 individuals were excluded for the PM_2.5_ analyses). Exposure was calculated by averaging daily PM_2.5_ concentrations for each individual over the prenatal period (calculated as described for BC above) and was modeled as a continuous variable. Because PM_2.5_ was modeled from 2000–2008, newborns born between April and December 1999 (30% of the cohort) had missing PM_2.5_ data.

#### 2.2.2. Immune Outcome Assessment

An immune outcome assessment has been reported previously [18]. Briefly, clinicians collected cord blood samples by needle/syringe from the umbilical vein after delivery. CBMCs were isolated from umbilical cord blood within 24 h without freezing and diluted to a concentration of 5 × 10^6^ cells/mL in 10% human serum.

Cytokines: The cytokine panel includes major pro-inflammatory (TNF-α and IL-6) and anti-inflammatory (IL-10) cytokines. CBMCs were incubated in triplicate [31], either unstimulated (media alone) or treated with one of three immune system stimulants (see below), and cultured for 72 h. Cell supernatants were analyzed for IL-6, IL-10, and TNF-α production using enzyme-linked immunosorbent assay (Endogen, Rockford, IL, USA), following the manufacturer’s protocol. Assay sensitivities were <3 pg/mL for IL-10 and <2 pg/mL for TNF-α. The percent of samples below the limit of detection varied from 0% to 14.5%.

Stimulated cytokines: Cell aliquots were stimulated with either 5 μg/mL phytohemagglutinin (PHA), 30 μg/mL cockroach extract (Bla g 2), or 30 μg/mL house dust mite extract (Der f 1). PHA is a mitogen that activates lymphocytes in a way that closely mimics antigenic stimulation of immune-competent cells in vitro [32,33,34]. Response to PHA does not depend on antigen-presenting cells. Bla g 2 and Der f 1 are common environmental allergens used in many studies to stimulate adaptive immune cells in vitro [35,36]. Prior work has shown that CBMCs respond to these extracts by proliferating and secreting cytokines. This response has been interpreted as a measure of the adaptive immune system’s competence to react to antigen [37].

#### 2.2.3. Depression Assessment

Assessment of maternal depression has been described previously [18], and was evaluated as follows:

Women were defined as having probable depression in pregnancy if they reported high symptoms and/or were prescribed antidepressants during pregnancy. Mothers reported depressive symptoms during the past seven days at the mid-pregnancy visit using the Edinburgh Postnatal Depression Scale (EPDS), a 10-item questionnaire that has been validated for use in pregnancy [38,39]. Responses are on a Likert scale ranging from 1 (most of the time) to 4 (not at all); responses are scored such that a higher total score indicates more symptoms of depression. A cut-off score of greater than 12 for probable clinical depression in pregnancy has been validated in this and other large cohorts [38,39,40]. Antidepressant use during pregnancy was determined from electronic medical records: a list of medications prescribed during pregnancy was compiled, and antidepressants were identified by a physician. We defined women who had an EPDS score > 12, and women who were prescribed antidepressants during pregnancy, regardless of their EPDS score, as having probable depression during pregnancy.

Pre-pregnancy depression history was assessed using three questions included on the mid-pregnancy questionnaire. Participants were asked an initial screening question: “Before this pregnancy, was there ever a period of time when you were feeling depressed or down or when you lost interest in pleasurable activities most of the day, nearly every day, for at least 2 weeks?” Women who responded affirmatively were asked two follow-up questions: (1) “Before this pregnancy, did you ever see a health care professional who said that you were depressed?” and (2) “Before this pregnancy, did a health care professional ever prescribe a medication for you for depression?” We defined women as having a history of depression if they responded affirmatively to the screener question and one or both of the follow-up questions.

Depression is a recurring condition [19,20], and its health effects are considered to be the result of cumulative burden rather than acute exposure [21]. We, therefore, characterized women as ever depressed (yes/no) if they scored as having either probable depression in pregnancy or a history of depression, or both, and used this as our primary depression exposure. The reference group was women who experienced depression neither before nor during pregnancy. Of the 463 women in the final sample, 34 had probable depression during pregnancy without a reported history of depression, 27 only had a pre-pregnancy history of depression, and 19 had both probable depression during pregnancy and a history of depression (see sample flow chart, Figure S1).

#### 2.2.4. Covariate Assessment

Women reported their age, race/ethnicity, household income, education level, smoking history, history of hypertension, and maternal and paternal history of asthma and atopy at enrollment, by interview and questionnaire. Mothers self-reported pre-pregnancy weight and height, from which we calculated body mass index (BMI, kg/m^2^), which has been shown to affect cord blood immune parameters [41]. We obtained the child’s birth date and sex from the hospital delivery record. Participants reported cigarette use during pregnancy by questionnaire (mid-pregnancy) and interview (delivery).

### 2.3. Statistical Analyses

Because the cytokine distributions were right-skewed, we ln-transformed them for analyses. PM_2.5_ and BC were normally distributed, so the data were not transformed. We tested the main effects of air pollution on cytokine concentrations and then tested for potential effect modification by depression. The main effects of depression were examined in a previous study [18]. We used Chi-square, independent T, or ANOVA tests to compare covariate distributions across levels of categorical air pollution variables or to compare means for continuous air pollution variables across levels of each covariate. We used separate multivariate regression models to assess relationships of air pollution with each outcome, including IL-10, TNF-α, and IL-6 after incubation without stimulant (MED) and after stimulation with PHA, Bla g 2, or Der f 1 extract.

In general, we used linear regression models to assess associations of immune outcomes with air pollution and potential effect modification by depression status. However, for unstimulated IL-10, 14.5% of samples fell below the level of detection of the assay. For this outcome, we used Tobit regression (using the PROC QLIM procedure in SAS) as it adjusts for censored data by combining a probit model to account for a fraction of samples censored, with a truncated regression model for those outcome values that score above the censored cut-off.

We tested a series of 3 separate main effect models for each combination of air pollution and immune outcome. Model 1 was minimally adjusted for child sex, the season of birth, and the year of birth. Model 2 included baseline demographic covariates, while Model 3 included the behavioral covariates. Maternal and paternal history of atopy was not associated with any immune outcomes in initial models and were not included further.

We tested independent effects of air pollution and mother’s depression by adding depression to the main effects models. We then tested potential effect modification of air pollution by depression using separate models that included an interaction term (air pollution x depression status). We also ran models stratified by ever and never depressed to explore potential interactions in more detail. To increase the interpretability of the regression coefficients, since immune outcomes were log-transformed in our models, we exponentiated coefficients to obtain differences in the ratio of the expected geometric means of the outcome variable.

As secondary analyses, we considered potential short-term effects of all air pollution exposures, including 7-day and 14-day averages before delivery date (see Supplemental Material for methods and results).

Missing data: 135 women (29%) were missing data on covariates or depression (Table S2 compares those with and without data on depression). To reduce bias and improve model precision, we imputed missing depression and covariate values using a chained equation multiple imputation model (PROC MI in SAS). Imputed values were derived using the full Project Viva cohort (*n* = 2128); the imputation model included exposure and outcome variables, all covariates, and other potential predictors of the missing data [42]. Following prior work in Project Viva [18,27] and considering recommendations for when the fraction of missing values is relatively high [43], we generated 50 imputed data sets and combined them using PROC MIANALYZE [44]. Final models included participants with imputed depression and covariate data. Air pollution data were not imputed. Participants with missing measurements of an outcome for a given exposure-outcome analysis were excluded from that analysis.

We performed our analyses using SAS Version 9.4 (SAS Institute Inc., Cary, NC, USA).

## 3. Results

Compared to those in the study sample, women excluded from the sample (those without cord blood measures for cytokines, *n* = 1665) were more likely to be Black or Hispanic (see Table S1). Air pollution measures were moderately inter-correlated (Table S3: r = 0.35–0.40; except BC with traffic density: r = 0.57). Figure S2 shows the distribution of cytokine measurements obtained from unstimulated CBMCs, and from CBMCs stimulated with mitogen (PHA) and allergens (Bla g 2 and Der f 1).

Table 1 shows the mean air pollution exposures by characteristics of the participants in the study (overall *n* = 463). PM_2.5_ varied minimally by levels of exposure, with the exception of seasonality. For all other pollutants, women who were lower-educated or Black had higher mean exposure than White women. Those with higher exposure to traffic or BC had higher levels of depression.

**Figure 1:**
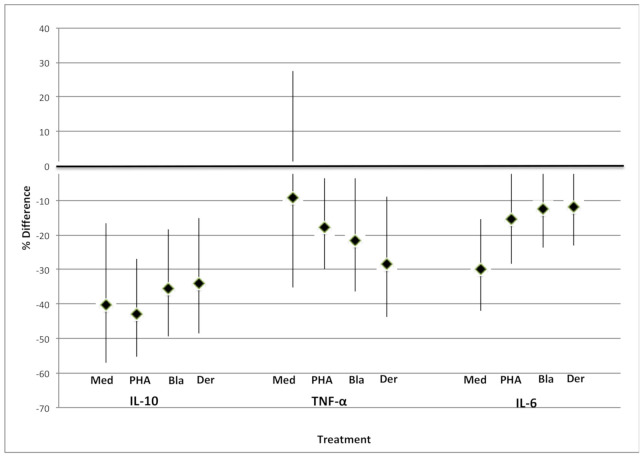
Percent difference in cord blood cytokine concentrations per μg/m^3^ increase in PM_2.5_ exposure. Unstimulated (Med) IL-10 was analyzed using Tobit regression; others were analyzed using linear regression. Models were adjusted for maternal age, race/ethnicity, education, household income, child sex, the season of birth, pre-pregnancy BMI, and smoking. PM_2.5_ exposure was averaged over pregnancy.

Considering effects of each type of air pollution on cytokine concentrations, findings were strongest for PM_2.5_ (Figure 1). PM_2.5_ exposure averaged over pregnancy showed robust inverse associations with all cytokines (unstimulated and stimulated). For example, in cells stimulated with Bla g 2, IL-10 decreased by 36% (95% CI = −49%, −18%) per 1 μg/m^3^ increase in PM_2.5_ and IL-6 decreased 13% (95% CI = −24%, 0.2%). All associations were statistically significant at *p* < 0.10 except for unstimulated levels of TNF-α. Associations for other air pollution measures, including proximity to a roadway, traffic density, and BC, were inconsistent across stimulants, often close to null and mostly non-significant (Figure S3).

When we added mother’s depression scores to the models that included PM_2.5_ (Table 2), air pollution effect estimates for PM_2.5_ did not appreciably change. For IL-10 only, effects of PM_2.5_ and depression appeared to be additive, as depression had a significant, independent effect on cytokine concentrations.

**Figure 2:**
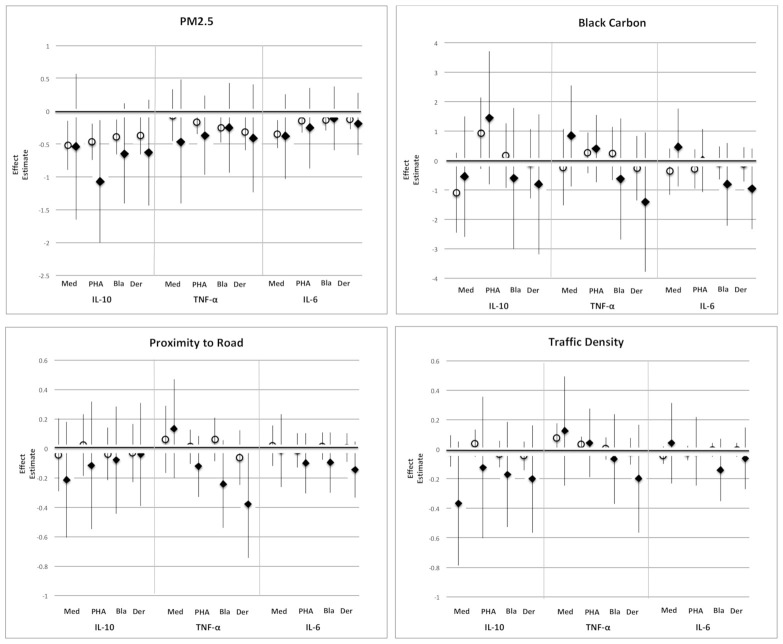
Effect estimates * for the association of air pollution and cytokine concentrations (in unstimulated (Med) cord blood lymphocytes or for those stimulated with phytohemagglutinin (PHA), cockroach antigen (Bla), or dust mite antigen (Der)), in ever and never depressed women. Open circles: never-depressed mothers. Closed diamonds: ever-depressed mothers. Unstimulated IL-10 was analyzed using Tobit regression; TNF-α and IL-6 were analyzed using linear regression. Outcomes were log-transformed for analysis. Models were adjusted for maternal age, race/ethnicity, education, household income, child sex, the season of birth, pre-pregnancy BMI, and smoking. * For PM_2.5_ and BC, (effect estimate × 100) is approximately the % change in cytokine concentration for a 1-unit increase in air pollution. For proximity to road and traffic density, the effect estimate is the % change in cytokine concentration for a 1% increase in air pollution.

We found no significant evidence that depression modified the association of pollution with any of the cytokine responses (See Table S4, interaction terms). However, in stratified analyses, although confidence intervals were large, effect estimates for the ever-depressed group tended to be of larger magnitude and more negative than for the never-depressed group for most air pollution exposures (see Figure 2). These findings hint that among more versus less depressed women, exposure to air pollution might be associated with even lower cytokine levels.

In analyses examining the relationships of short-term (averages of 7 and 14 days before birth) PM_2.5_ and BC with all outcomes, with two exceptions, we found statistically null results with small effect sizes. Stronger associations were evident for PM_2.5_ averages in the 7 days (β = 0.07; 95% CI = 0.02, 0.13) and 14 days (β = 0.07; 95% CI = 0.00, 0.14) before birth with unstimulated TNF-α (MED); and for PM_2.5_ 14-day averages with TNF-α stimulated with cockroach antigen (β = −0.06; 95% CI = −0.11, 0.01) (see Supplemental Material for methods and results of these analyses).

## 4. Discussion

In a cohort of over 400 women, we found that CBMC production of the predominantly pro-inflammatory cytokines IL-6 and TNF-α and of the anti-inflammatory cytokine IL-10 were all lower among mothers exposed to higher levels of PM_2.5_ during pregnancy. Adding mother’s depression scores to the model had an additive effect on this decrease in cytokine concentrations in relation to IL-10. While no effects of other measures of air pollution exposure on these cytokines were evident in the main effect analyses, when stratified by mother’s experience of depression, a suggestive pattern emerged with cytokine levels often lower in the ever- versus never-depressed group across all measures of air pollution. However, confidence intervals were wide, and interaction terms between mother’s depression and air pollution exposure were rarely significant (see Table S4).

Based on previous work linking air pollution exposure to an increase in inflammatory processes and biomarkers [3,45,46,47], we hypothesized that increased exposure to air pollution during pregnancy would lead to higher concentrations of pro-inflammatory cytokines and lower concentrations of anti-inflammatory cytokines assayed from neonates’ CBMCs. Instead, we observed an overall lower level of all cytokines associated with higher exposure to PM_2.5_. Our findings may differ from many prior studies in part because we considered effects in neonates, whereas most studies examined outcomes in childhood or adulthood.

Research examining gestational exposure to air pollution and offspring immune responses at birth is sparse [48]. A recent animal study found that mice with gestational exposure to ultrafine particulates (aerodynamic diameter < 0.1 μm) had reduced inflammatory response to house dust mite antigen in offspring when challenged from zero to four weeks of age [7]. This is consistent with our results. Results from the few human studies that examined biomarkers at birth are inconsistent. A study of 4450 neonates in the Generation R cohort from Rotterdam [49] found elevated levels of C-reactive protein, an inflammatory biomarker, in cord blood from mothers who scored in the highest quartile of PM_10_ exposure averaged over pregnancy, suggesting that prenatal PM_10_ exposure may promote fetal inflammation. In contrast, a study more directly comparable with ours assessed the unstimulated in vitro expression of the pro-inflammatory cytokines IL-6, TNF-α, and interleukin 1β, as well as of the anti-inflammatory cytokine IL-10, from cryopreserved cord blood of 265 healthy neonates, in relation to several measures of prenatal air pollution exposure [50]. That study found no overall association between cytokine concentrations and living near major roads or exposure to PM_10_ during pregnancy; however, higher PM_10_ exposure during the last three months of pregnancy was associated with elevated cord blood IL-1ß levels and higher exposure during the last week of pregnancy was suggestively associated with lower IL-6 and IL-10 levels. This study aligns with our robust observation of lower levels of IL-6, IL-10, and TNF-α with increased exposure to PM_2.5_ averaged across pregnancy, although we did not observe such effects in association with short-term PM_2.5_ or BC exposure in the 7 or 14 days before birth. None of these prior studies of air pollution and neonate immune function considered potential effect modification by levels of maternal depression.

A handful of human studies have examined shifts in T-cell fractions in cord blood in relation to exposure to air pollution during pregnancy. Because these studies assessed the percentage of cells of each type rather than absolute numbers of cells and did not measure cytokines produced by the cells, they are not directly comparable to our findings. However, one study measured exposure to PM_2.5_ and polycyclic aromatic hydrocarbons (PAH) in 1397 pregnant women in the Czech Republic and found short-term exposure to PAH or PM_2.5_ in the 14 days before birth was associated with lower total T-lymphocytes (CD3+), T-helper cells (CD4+), and killer cells (CD8+) from cord blood collected at delivery [10]. A study from France found similar results [9]. These results are consistent with our finding of a global lowering across both anti- and pro-inflammatory cytokines with higher prenatal exposure to PM_2.5_ (Figure 1).

In the present study, mother’s depression and PM_2.5_ exposure were independent predictors of IL-10 concentrations (Table 2). When we stratified by mother’s experience of depression, although error bars were wide and interaction terms were rarely significant (Table S3), often the associations between air pollution measures and cytokine concentrations were more negative in neonates of ever- versus never-depressed mothers (Figure 2). Although one must be cautious when interpreting imprecise effects in relatively small samples, and more work needs to be done in larger and more economically diverse cohorts, these observations are in line with a growing body of research motivated by the realization that more socially disadvantaged individuals are often disproportionately exposed to environmental contaminants such as air pollution [15]. Some investigators have suggested that this creates “double jeopardy” whereby high levels of distress may increase susceptibility such that exposure to environmental contaminants will have adverse health effects at a relatively lower dose [16].

Studies in animals and humans have begun to test the hypothesis of double jeopardy. For example, an experimental animal study in which female mice were exposed to both air pollution (diesel exhaust particles) and stress during gestation found that their adult offspring showed higher anxiety and (in males only) lower cognition than offspring of those mice exposed to air pollution alone [51]. Among humans, much of the work evaluating this double jeopardy hypothesis has considered the interplay of stress-related psychosocial factors, ranging from socioeconomic position to demoralization, in conjunction with air pollution, on early life health [52]. For example, a study from a Mexico City birth cohort found that exposure to PM_2.5_ during the first trimester was associated with increased wheeze among children at 48 months of age, but only in children of women who reported high versus low stress on an index of negative life events [17].

A joint effect of prenatal air pollution exposure and maternal depression is biologically plausible considering that psychosocial stress and environmental toxicants, including air pollution, appear to share common mechanisms on health, not only through immune pathways but through effects on oxidative stress, the hypothalamic-pituitary-adrenal system, and the sympathetic nervous system [53,54]. Studies in newborns of depressed pregnant mothers have found differences in neurotransmitter and hormone patterns that indicate potential dysregulation of the infant’s HPA axis [55], and we previously found in this cohort that maternal prenatal depression was associated with lower levels of IL-10 in cord blood at birth [18]. In animals, experimental studies that examine the health effects of gestational exposure to air pollution on adult offspring also implicate immune pathways and inflammatory processes. For example, in a mouse model of bronchial asthma, researchers found a significant increase in inflammatory cells in bronchoalveolar lavage fluid from 5-week and 10-week old offspring of mothers exposed to fine and ultrafine particulate matter during pregnancy; these findings were even more marked in adult (15 and 30 weeks) offspring upon exposure to an allergen [6]. Such immune dysregulation has been implicated as a possible mechanism through which gestational exposure to air pollution might disrupt neurodevelopment [11].

Our finding that gestational exposure to air pollution is linked to global decreases in cytokine responses at birth begs the question of whether cytokine levels measured at birth have an effect on health across the course of the child’s life. Some studies have found lower levels of cord blood cytokines associated with a greater likelihood of adverse child health outcomes such as wheeze, eczema, and other allergic disorders [56], supporting the idea that decreases in cytokine responses at birth are related to later health [57]. In Project Viva, a study of 446 mother-child pairs found that lower levels of cord blood IFN-γ were associated with a higher risk of acute lower respiratory infection in the first year of life [58]. Follow-up has continued for the Project Viva children, offering the opportunity to extend these findings to examine associations of neonatal immune responses with participants’ health throughout childhood.

Our study has limitations. Exposure misclassification in air pollution measures and depression is possible. We did not individually test all possible components of air pollution that might lead to inflammation, using distance to roadway and traffic density estimates as proxies for long-term traffic-related ambient air pollution exposure. Evaluating the effects of other air pollution components at a more granular level is an important direction for future research. BC and PM_2.5_ values were modeled rather than being measured at the individual level; we only had geographic information for the mothers’ residential address during pregnancy, so we could not assess air pollution exposure before pregnancy. Depressive symptoms were only measured once during pregnancy. However, if the timing is important, exposure misclassification due to experience of depression earlier or later in pregnancy would likely bias results towards the null. We could not fully account for a history of inflammatory processes among the women in our sample; however, few women (*n* = 4) reported a history of diabetes, and other inflammatory diseases are likely rare in this population (e.g., inflammatory bowel disease has a prevalence well below 1% in pregnant women [59,60]). Finally, the study cohort is composed of mostly white, well-educated mothers and their children residing in Eastern Massachusetts. The numbers of women in our study experiencing high levels of both exposures were small, so our power to detect a statistically significant interaction was limited. Conducting such studies in more socially disadvantaged populations may provide more insight into how double jeopardy might contribute to social disparities in health.

Our study has several strengths, as well. To the best of our knowledge, this is the first study to examine the joint effect of air pollution and maternal depression on offspring immune responses at birth. Exposures were assessed prospectively, and a variety of valid measures of prenatal depression were available. PM_2.5_ and BC were measured using validated land-use regression models based on GIS to estimate exposures at the mother’s residential address. In addition, measurements were available that made it possible to account for a broad array of potential confounders and covariates.

## 5. Conclusions

Over the past decade or more, separate lines of research have documented adverse health effects on offspring of mothers exposed to environmental or psychosocial risk factors before birth. Our findings that higher gestational PM_2.5_ exposure is associated with lower neonatal immune responses, along with a suggestive, although not statistically significant, pattern of larger effects of air pollution on immune responses of newborns with depressed mothers, point to the need for further studies that consider the social context when evaluating effects of gestational exposure to air pollution on health outcomes in the child. This is particularly true in vulnerable communities where psychosocial stressors and higher exposures to environmental toxicants are likely to coincide.

## Figures and Tables

**Table 1:** Air pollution means ± standard errors according to participant characteristics.

Characteristic	Overall *n* (%)	PM_2.5_^a,b^	Distance to Road ^c^	Traffic ^d^	Black Carbon ^b^
	(*n* = 463)	(*n* = 322)	*n* = 461	*n* = 459	*n* = 455
**Mother**					
Household income ≤ $40,000/year					
No	363 (78)	*12.1 ± 0.1*	**1107.7 ± 1.1**	**498.8 ± 1.1**	**0.69 ± 0.01**
Yes	54 (12)	*12.3 ± 0.1*	**742.5 ± 1.2**	**1056.0 ± 1.2**	**0.80 ± 0.03**
Education ≤ high school					
No	415 (90)	12.0 ± 0.1	1074.9 ± 1.1	**508.9 ± 1.1**	**0.69 ± 0.01**
Yes	46 (10)	12.1 ± 0.1	820.6 ± 1.3	**1144.0 ± 1.2**	**0.81 ± 0.03**
Race/ethnicity					
Black	62 (13)	12.1 ± 0.1	**544.6 ± 1.2**	**927.3 ± 1.1**	**0.82 ± 0.02**
Hispanic	27 (6)	12.2 ± 0.2	871.3 ± 1.4	686.9 ± 2.1	**0.85 ± 0.03**
Other	43 (9)	12.0 ± 0.1	**862.6 ± 1.2**	766.8 ± 1.2	0.75 ± 0.03
White	329 (71)	12.0 ± 0.1	**1236.5 ± 1.1**	**469.8 ± 1.2**	**0.67 ± 0.01**
Pre-pregnancy BMI, kg/m^2^					
Underweight, <18.5	19 (4)	12.4 ± 0.3	**614.0 ± 1.4**	946.0 ± 1.2	0.76 ± 0.05
Healthy weight, 18.5−<25	275 (59)	12.1 ± 0.1	**1053.6 ± 1.1**	469.8 ± 1.2	0.69 ± 0.01
Overweight, 25−<30	98 (21)	11.9 ± 0.1	**1339.4 ± 1.1**	634.1 ± 1.2	0.71 ± 0.02
Obese, >30	69 (15)	12.2 ± 0.1	**845.6 ± 1.2**	766.8 ± 1.2	0.75 ± 0.03
Age < 23 years					
No	436 (94)	12.0 ± 0.1	1074.9 ± 1.1	**535.0 ± 1.1**	**0.70 ± 0.01**
Yes	27 (6)	12.3 ± 0.2	706.3 ± 1.3	**974.8 ± 1.3**	**0.81 ± 0.04**
Pregnancy smoking status					
never	322 (70)	12.1 ± 0.1	1022.5 ± 1.1	503.8 ± 1.2	*0.70 ± 0.01*
former	82 (18)	11.9 ± 0.1	1141.4 ± 1.2	579.5 ± 1.2	*0.67 ± 0.02*
pregnancy	54 (12)	12.2 ± 0.1	1043.1 ± 1.2	**899.9 ± 1.2**	*0.77 ± 0.04*
Cesarean delivery					
No	370 (80)	12.1 ± 0.1	1053.6 ± 1.1	573.8 ± 1.1	*0.71 ± 0.01*
Yes	87 (19)	12.0 ± 0.1	1096.6 ± 1.2	438.0 ± 1.3	*0.67 ± 0.02*
Probable depression in pregnancy					
No	308 (67)	12.1 ± 0.8	1152.9 ± 3.5	**455.9 ± 14.6**	**0.68 ± 0.21**
Yes	53 (11)	12.0 ± 0.7	1074.9 ± 3.6	**766.8 ± 4.0**	**0.76 ± 0.26**
Ever depressed					
No	281 (61)	12.1 ± 0.1	1152.9 ± 1.1	**433.7 ± 1.2**	**0.68 ± 0.01**
Yes	80 (17)	12.1 ± 0.1	1096.6 ± 1.2	**782.3 ± 1.2**	**0.75 ± 0.03**
**Child**					
Season of birth					
Winter	116 (25)	**11.7 ± 0.1**	1043.1 ± 1.1	420.8 ± 1.3	**0.73 ± 0.02**
Spring	118 (25)	**12.1 ± 0.1**	1224.1 ± 1.1	474.5 ± 1.3	**0.73 ± 0.02**
Summer	123 (27)	**12.3 ± 0.1**	1074.9 ± 1.1	615.4 ± 1.2	**0.70 ± 0.02**
Fall	106 (23)	**12.2 ± 0.1**	862.6 ± 1.2	798.1 ± 1.2	**0.65 ± 0.02**
Child sex					
Male	253 (55)	*12.0 ± 0.1*	1043.1 ± 1.1	603.2 ± 1.1	0.72 ± 0.01
Female	210 (45)	*12.1 ± 0.1*	1053.6 ± 1.1	503.8 ± 1.2	0.69 ± 0.01

^a^ PM_2.5_: particulate matter < 2.5 microns; ^b^ Calculated by averaging daily concentrations (pg/mL) for each individual over the prenatal period, calculated as the date of last menstrual period through the day before birth; ^c^ Measured as shortest distance in meters between the geocoded location of the mother’s reported residential address and a primary major roadway (US Census feature class A1 or A2); ^d^ Measured as the length of all roads (km) within 100 m of mother’s residence, multiplied by the traffic counts on those roads (vehicles/day); Bold: mean exposure differs by level of characteristic at *p* < 0.05; italics: mean exposure differs at *p* < 0.10 (ANOVA for categorical variables, chi-square for dichotomous variables).

**Table 2:** Percent change in neonatal cord blood cytokine concentration ^a^ associated with exposure to PM_2.5_^b^ and maternal depression ^c,d^.

Cytokine	Exposure	Main Effect, PM_2.5_^e^	Main Effects, Additive Model ^f^
		% Change (95% CI)	% Change (95% CI)
IL-10	Med ^g^		
	PM_2.5_	−40.12 (−57.10, −16.61)	−40.17 (−57.07, −16.61)
	Ever depressed		0.26 (−52.70, 112.53)
	PHA		
	PM_2.5_	−42.83 (−55.36, −26.79)	−41.75 (−54.46, −25.49)
	Ever depressed		−44.3 (−66.62, −7.07)
	Bla		
	PM_2.5_	−35.59 (−49.3, −18.18)	−34.50 (−48.39, −16.88)
	Ever depressed		−42.34 (−65.05, −4.86)
	Der ^b^		
	PM_2.5_	−33.85 (−48.52, −14.99)	−32.99 (−47.85, −13.91)
	Ever depressed		−34.56 (−60.57, 8.61)
TNF	Medium		
	PM_2.5_	−9.16 (−35.23, 27.41)	−10.16 (−36.00, 26.12)
	Ever depressed		39.9 (−31.01, 183.66)
	PHA		
	PM_2.5_	−17.77 (−29.92, −3.52)	−17.1 (−29.36, −2.72)
	Ever depressed		−22 (−44.33, 9.29)
	Bla		
	PM_2.5_	−21.68 (−36.34, −3.65)	−21.02 (−35.82, −2.81)
	Ever depressed		−23.44 (−51.05, 19.76)
	Der		
	PM_2.5_	−28.45 (−43.74, −9.00)	−27.77 (−43.21, −8.12)
	Ever depressed		−27.38 (−59.10, 28.94)
IL-6	Medium		
	PM_2.5_	−29.95 (−42.06, −15.31)	−30.16 (−42.26, −15.51)
	Ever depressed		9.53 (−27.43, 65.31)
	PHA		
	PM_2.5_	−15.33 (−28.29, −0.02)	−14.97 (−28.03, 0.46)
	Ever depressed		−12.49 (−38.38, 24.3)
	Bla		
	PM_2.5_	−12.52 (−23.60, 0.17)	−12.30 (−23.45, 0.46)
	Ever depressed		−8.25 (−30.88, 21.78)
	Der		
	PM_2.5_	−11.76 (−23.13, 1.29)	−11.52 (−22.95, 1.61)
	Ever depressed		−8.99 (−32.06, 21.91)

^a^ Cytokine concentration was measured in pg/mL; ^b^ Per 1μg/m3 increase, averaged over pregnancy; ^c^ Ever vs. never depressed; ^d^ Models were adjusted for maternal age, race/ethnicity, education, household income, child sex, the season of birth, pre-pregnancy BMI, and smoking; ^e^ Model does not contain a term for maternal depression; ^f^ Model is adjusted for both PM_2.5_ and maternal depression; ^g^ Analyzed using Tobit regression; all others analyzed using linear regression.

## Data Availability

Project Viva protocols include a data sharing plan. The process for requesting data is detailed in the study policy document, available on the Project Viva website: https://www.hms.harvard.edu/viva/policies-for-using-our-data.pdf (accessed on 10 May 2021).

## Supplementary Materials

The following are available online at https://www.mdpi.com/article/10.3390/ijerph18105062/s1, Table S1: Participant characteristics according to whether excluded from or included in the Project Viva study sample for each air pollution exposure, Table S2: Participant characteristics according to depression status in the Project Viva study sample, Table S3: Spearman correlation coefficients for correlations between air pollution measures for the Project Viva study sample, Table S4: Interaction term (air pollution × ever depressed) for the joint association of air pollution and mother ever depressed before birth with cytokine concentrations from neonatal cord blood, Figure S1: Flow chart of sample sizes for those included in the study, Figure S2: Boxplots showing the production of cytokines in CBMCs, unstimulated (Med), and after stimulation with mitogen (PHA) and allergen (Bla g 2, Der f 1). (A) IL-10, (B) TNF-α, (C) IL-6, Figure S3: Effect estimates for the difference in cord blood cytokine concentrations with differences in proximities to roads, traffic exposure, or black carbon during pregnancy, Methods, and Results: Temporally resolved BC and PM_2.5_ measures.
