# Supplementary material for: Air Pollution, Neonatal Immune Responses, and Potential Joint Effects of Maternal Depression
Source: Int J Environ Res Public Health. 2021 May 11;18(10):5062. doi: 10.3390/ijerph18105062 (PMC8150899; doi:10.3390/ijerph18105062)
Supplement: Supplementary file 1 [file ijerph-18-05062-s001.zip › ijerph-969803-supplementary.pdf]

Air Pollution, Neonatal Immune Responses, and Potential Joint Effects of Maternal Depression

Jill Hahn, Diane R. Gold, Brent A. Coull, Marie C. McCormick, Patricia W. Finn, David L. Perkins, Sheryl L. Rifas Shiman, Emily Oken and Laura D. Kubzansky

Table S1. Participant characteristics according to whether excluded from (N=1665) or included in the Project Viva study sample (N=463) for each air pollution exposure (%, unless otherwise indicated).

Characteristic	Traffic Exposures		PM2.5		Black Carbon	
	Excluded	Included	Excluded	Included	Excluded	Included
	N=1651	N=461	N=1063	N=322	N=1625	N=455
Mother						
Pre-pregnancy BMI, kg/m^2: Mean ±SD	24.9 ±5.6	24.7 ±5.3	25.0 ±.6	24.5 ±4.8	24.9 ±5.6	24.7 ±5.3
Age, year: Mean ±SD	31.8 ±5.3	31.9 ±5.0	31.8 ±5.3	32.1 ±5.0	31.8 ±5.3	31.8 ±5.0
Depression score: Mean ±SD	5.3 ±4.7	5.3 ±5.0	5.3 ±4.8	5.16 ±4.57	5.3 ±4.7	5.2 ±5.0
Race/ethnicity						
Black	17	14	17	12	17	13
Hispanic	8	6	8	6	8	6
White	65	72	65	72	65	71
Other	10	9	10	10	10	9
Education ≤high school	13	10	13	11	13	10
Household income ≤$40,000/year	17	12	16	13	16	13
Pregnancy smoking status				0		0
Never	68	70	68	71	68	70
Former	19	18	19	17	19	18
Smoked during pregnancy	13	11	13	11	13	12
Cesarean delivery	25	19	25	18	25	19
Score of >12 on EPDS	8	11	9	9	9	10
Pre-pregnancy depression history	11	12	11	14	11	12
Antidepressants prescribed during pregnancy	3	4	3	4	3	4
Ever depressed	20	22	20	22	20	22
Child						
Season of birth						
Winter	25	24	24	28	24	25
Spring	26	26	26	26	26	26
Summer	27	27	28	24	27	27
Fall	22	23	22	22	22	23
Child sex female	49	45	49	45	49	45

[a]Traffic exposures include proximity to major road and near-residence traffic density.

Table S2. Participant characteristics according to depression status in the Project Viva study sample (N=463).

	Depression		
	**Missing N=102**	**Never N=281**	**Ever N=80**
**<u>Mother</u>**			
Pre-pregnancy BMI, kg/m^2 (*mean ± SD)*	26.9 (6.3)	23.9 (4.8)	24.72 (5.0)
Age, years (*mean ± SD)*	29.7 (5.5)	32.5 (4.5)	31.9 (5.6)
Race/ethnicity			
Black	30 (29)	24 (9)	8 (10)
Hispanic/Other	15 (15)	23 (8)	10 (12)
White	53 (52)	220 (78)	56 (70)
Education ≤high school	21 (21)	17 (6)	8 (10)
Household income ≤$40,000	17 (17)	24 (9)	13 (16)
Not married/partnered	18 (18)	10 (4)	8 (10)
Hypertensive disorders of pregnancy			
Gestational hypertension	6 (6)	13 (5)	10 (13)
Pre-eclampsia	5 (5)	7 (3)	1 (1)
Cesarean delivery	19 (19)	52 (19)	16 (20)
Smoking			
Former smoker	11 (11)	57 (20)	14 (18)
Smoked during pregnancy	16 (16)	21 (8)	17 (21)
**<u>Child</u>**			
Season of birth			
Winter	31 (30)	56 (20)	29 (36)
Spring	24 (24)	80 (29)	14 (18)
Summer	21 (21)	78 (28)	24 (30)
Fall	26 (26)	67 (24)	13 (16)
Child sex female	37 (36)	138 (49)	35 (44)

Table S3. Spearman correlation coefficients (N) for correlations between air pollution measures for Project Viva study sample (N=463).

Air Pollution Measure	Proximity to Roadway	Traffic Density	PM2.5
Traffic Density	0.35 (459)		
Prenatal PM2.5	0.36 (322)	0.35 (320)	
Prenatal Black Carbon	0.36 (455)	0.57 (455)	0.40 (318)

Table S4. Interaction term (air pollution x ever depressed) for the joint association of air pollution and mother ever (vs. never) depressed before birth with cytokine concentrations from neonatal cord blood.*.

		PM2.5	BC	Proximity	Traffic Density
**Cytokine/Stimulant**		**β (95% CI)**	**β (95% CI)**	**β (95% CI)**	**β (95% CI)**
IL-10	Medium	1.01 (0.33, 3.07)	0.93 (0.08,11.39)	0.96 (0.57, 1.61)	0.73 (0.47, 1.14)
	PHA	0.59 (0.25, 1.40)	1.69 (0.17,16.43)	0.94 (0.60, 1.49)	0.92 (0.62, 1.38)
	Bla g2	0.82 (0.38, 1.80)	0.64 (0.05, 7.71)	0.96 (0.63, 1.45)	0.91 (0.64, 1.29)
	Der f1	0.83 (0.37, 1.89)	0.62 (0.04, 8.87)	1.00 (0.65, 1.55)	0.90 (0.61, 1.31)
TNF	Medium	0.64 (0.23, 1.78)	2.10 (0.20,21.92)	1.01 (0.65, 1.56)	0.97 (0.63, 1.48)
	PHA	0.88 (0.53, 1.46)	1.19 (0.32, 4.47)	0.86 (0.67, 1.10)	1.05 (0.85, 1.31)
	Bla g2	1.00 (0.52, 1.93)	0.57 (0.07, 4.58)	**0.71 (0.51, 0.99)**	0.96 (0.71, 1.29)
	Der f1	0.87 (0.42, 1.80)	0.65 (0.05, 7.87)	0.66 (0.41, 1.09)	0.88 (0.61, 1.26)
IL-6	Medium	0.91 (0.48, 1.71)	2.22 (0.48,10.19)	0.98 (0.75, 1.29)	1.05 (0.82, 1.36)
	PHA	0.86 (0.51, 1.46)	1.59 (0.46, 5.53)	0.92 (0.73, 1.17)	1.07 (0.86, 1.33)
	Bla g2	1.05 (0.70, 1.59)	0.55 (0.15, 2.05)	0.87 (0.70, 1.07)	0.91 (0.75, 1.09)
	Der f1	0.96 (0.63, 1.47)	0.61 (0.15, 2.55)	**0.83 (0.66, 1.03)**	0.95 (0.78, 1.16)

Note: Models were adjusted for maternal age (continuous), race/ethnicity, education (≤high school), household income (≤$40,000/year), child sex, season of birth (date, sine, and cosine of birthdate), pre-pregnancy BMI (continuous), and smoking (never, former, during pregnancy). Models using values from IL-10 in Medium were analyzed using Tobit regression, to account for proportion of samples below level of detection of assay. All other models were analyzed using linear regression. * β represents the ratio of geometric mean for one-unit increase in air pollution. Thus the estimate is considered statistically non-significant if the confidence interval includes 1. Bold text: p<0.10.

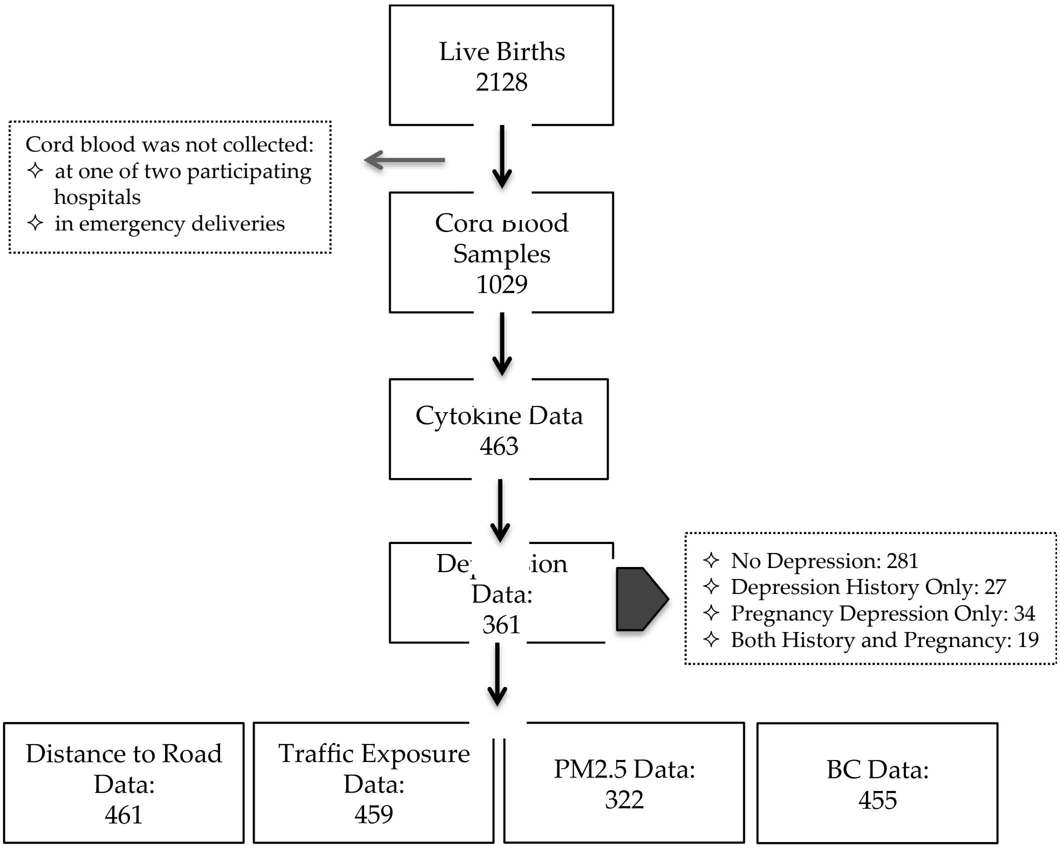

Figure S1. Flow chart of sample sizes for those included in the study. Note: missing depression and covariate data were imputed, using multiple imputation, for purposes of analyses. Missing air pollution data were not imputed.

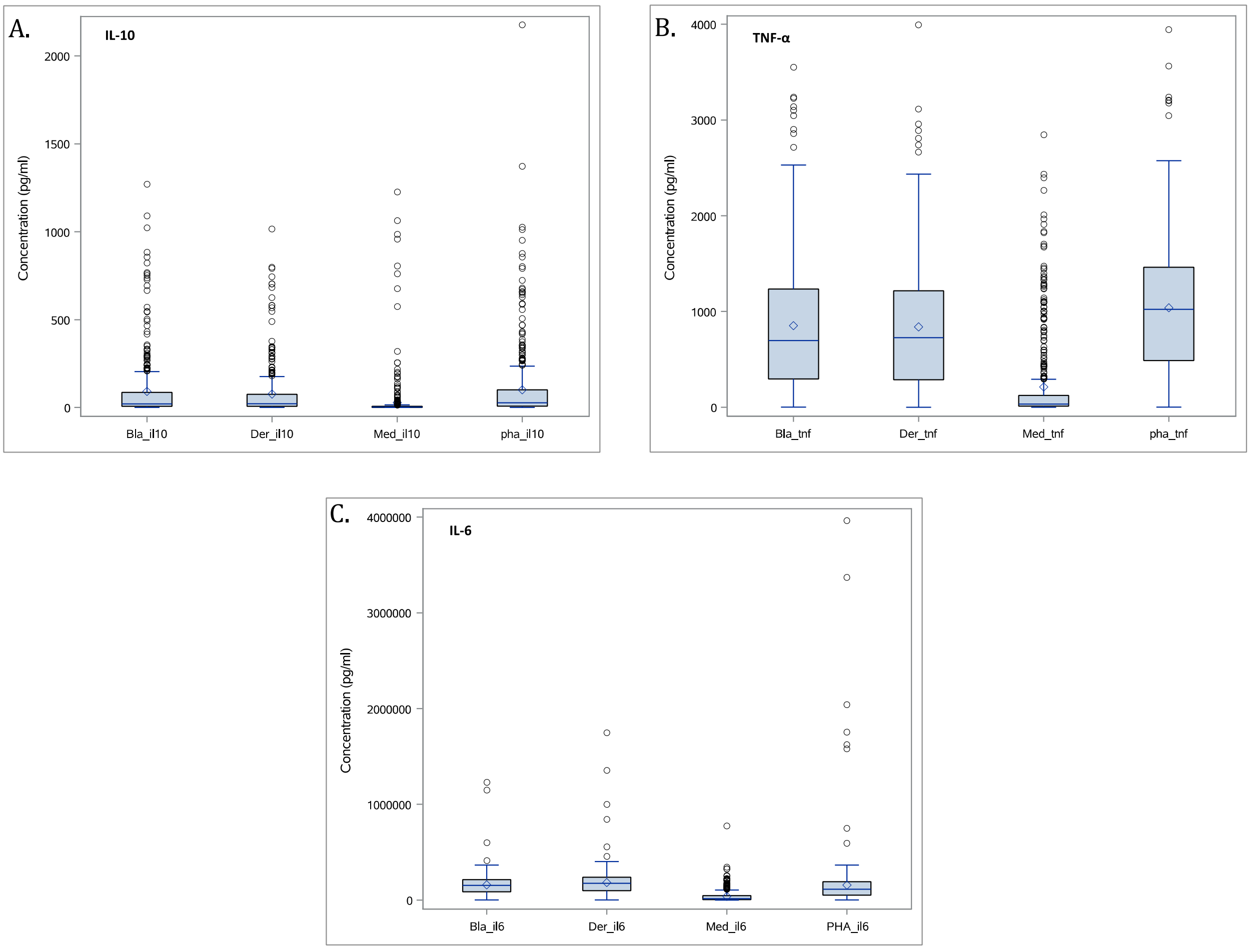

Figure S2. Boxplots: production of cytokines in cord blood mononuclear cells collected at delivery from Project Viva study participants (N=463), unstimulated (Med), and after stimulation with mitogen (PHA) and allergen (Bla g2, Der f1). (**A**) Interleukin-10 (IL-10), (**B**) Tumor necrosis factor α (TNF-α), (**C**) IL-6.

A.

B.

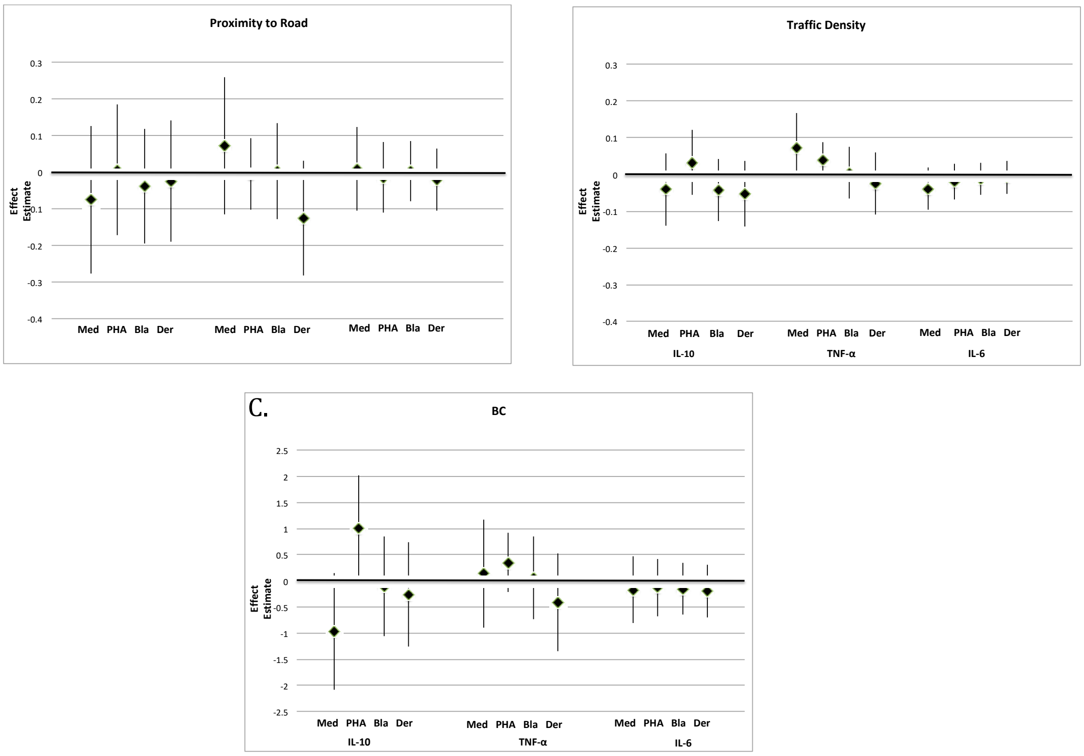

Figure S3. Effect estimates for the difference in cord blood cytokine concentrations with differences in proximity to road, traffic exposure, or black carbon during pregnancy. Cytokines: IL-10, TNF-*α*, and IL6 (pg/ml). Med: unstimulated lymphocytes. PHA: phytohemagglutin; Bla: cockroach antigen; Der: dust mite antigen. Unstimulated (Med) IL-10 was analyzed using Tobit regression; others were analyzed using linear regression. Models adjusted for maternal age, race/ethnicity, education, household income, child sex, season of birth, pre-pregnancy BMI, and smoking.

Temporally resolved BC and PM2.5 measures

Ambient concentrations of PM2.5 and BC were measured hourly at a central monitoring site (Harvard Supersite) in Boston. PM2.5 concentrations were measured with a tapered element oscillation microbalance (model 1400A; Rupprecht and Pastashnick, East Greenbush, NY), and BC concentrations were measured using an aethalometer (model AE-16; Magee Scientific Co., Berkeley, CA)(Kang et al. 2010). Missing hourly data for PM2.5 and BC were imputed, using long-term trend; season (1 = May–September, 0 = otherwise); seasonality (sine and cosine terms); hour of the day; day of the week; weather (barometric pressure, relative humidity, mean temperature, horizontal visibility, wind direction, and wind speed); and interactions with season, wind, and hour of the day. In total, 2% of the 24-hr PM2.5 and BC estimates were imputed.

Time windows of exposure ("moving averages") of 2, 7, and 14 days before birth were calculated to evaluate air pollution exposures close to the time of birth. For each central-site exposure period of interest, for each participant, we required that participants live within 40 km of the Harvard Supersite and that exposure data be available for at least 75% of the time in the specific averaging period; otherwise, the exposure was set to missing.

Statistical Analysis

Additional Analyses

We ran models of the same form as described above using 7-day, and 14-day before birth moving averages of spatio-temporally resolved estimates of BC and PM2.5, to assess possible short-term effects of maternal air pollution exposure on neonatal immune responses. In addition, we ran models of temporally resolved BC and PM2.5 exposure, to determine whether the modeled exposures gave results comparable to the spatio-temporally resolved exposures.

Results

We compared temporal-spatially modeled values for 2 day, 7 day, and 14 day exposure windows before birth, for both BC and PM2.5, with temporally measured values for the same exposure windows. We found, for each pollutant, that effect estimates for the modeled versus the temporally resolved values for each pollutant showed similar patterns. Most of the effect estimates were close to 0 and statistically insignificant. The only exception was the association of BC with unstimulated TNF-α (incubated in medium only), which was positive and statistically significant for both the spatio-temporally resolved (β=1.14; CI 0.25-2.03) and the temporally resolved models (β=1.42; CI 0.27-2.58).
